# Clinicians’ Experiences and Perspectives about a New Lung Cancer Referral Pathway in a Regional Health Service

**DOI:** 10.5334/ijic.7627

**Published:** 2024-04-04

**Authors:** Zulfiquer Otty, Sarah Larkins, Rebecca Evans, Amy Brown, Sabe Sabesan

**Affiliations:** 1 College of Medicine & Dentistry, James Cook University, Townsville, Australia; 2 Australian Institute of Tropical Health and Medicine, James Cook University, Townsville, Australia; 3 Townsville Cancer Centre, Townsville University Hospital, Townsville, Australia

**Keywords:** referral pathways, lung cancer, clinicians’ experiences, qualitative study

## Abstract

**Introduction:**

Development and implementation of the Townsville Lung Cancer Referral Pathway [TLCRP] aims to reduce delays and improve referral patterns of people with suspected lung cancer in north Queensland, Australia. Reported in this paper is the experiences and perspectives of general practitioners [GPs] and specialists of the TLCRP.

**Methods:**

This was a descriptive qualitative study nested within a larger project evaluating TLCRP, utilising a broader implementation science framework. In-depth, semi-structured interviews with GPs and specialists were conducted. An iterative, inductive thematic analysis of interview transcripts was used to derive key codes, then grouped into themes regarding participant experiences and perceptions.

**Results:**

Data analysis identified two major themes and several sub-themes. The major themes were variation in the uptake of TLCRP and enhancing coordinated care and communication.

**Discussion:**

Several enablers and barriers to implementing TLCRP were identified. Barriers to adaptation of TLCRP included lack of clinical time, resistance to changing referral patterns, lack of familiarity or experience with HealthPathways and technology issues.

**Conclusion:**

Emerging themes from this study may be used to reduce the barriers and improve uptake of TLCRP and other health care pathways in the local health service and may have wider relevance in other settings.

## Introduction

Lung cancer is a leading cause of mortality and morbidity worldwide [1]. Regional, rural, remote patients as well as Aboriginal and Torres Strait Islander patients have worse outcomes from lung cancer [2,3,4]. There are significant delays in diagnosis and management of lung cancer in rural patients [5,6]. Delays in care of people with suspected lung cancer often occur in the interface between primary care and the hospital [7]. Efficient and streamlined referral of patients with suspected lung cancer is an integral part of providing optimal care for these patients [8]. There is emerging evidence that use of online care pathways, such as HealthPathways is associated with improved referral quality from primary care, more timely access to secondary care and standardization of clinical management decisions by general practitioners (GPs) [9,10,11]. This is in line with the development of integrated care pathways as best practice in the literature [12,13,14].

In Queensland, Australia, the web-based portal, ‘HealthPathways’ is used as the point-of-care clinical practice guideline for guiding GPs on the management, and specialist referral, of various medical conditions [15,16]. ‘Townsville HealthPathways’ is the locally agreed, evidence-based clinical pathway portal used in the local health service area, Townsville Hospital and health service in north Queensland, which serves a considerable number of geographically dispersed, rural, remote and Aboriginal and Torres Strait Islander populations. Implementation of Townsville HealthPathways aims to reduce delays and improve quality of care for these patients. The clinical pathways are available to local clinicians via a password-protected web-based portal. The Townsville Lung Cancer Referral Pathway (TLCRP) was developed by the primary author, in consultation with local GPs and lung cancer specialists, and went online in 2019 (Supplementary material 1).

General practices in the health service region use an electronic referral system called Smart Referrals to refer patients to specialist clinics in the public hospital. The Townsville HealthPathways web portal is partly integrated within the Smart Referrals platform. Whenever a GP makes an electronic referral for a person with suspected lung cancer for specialist care, the TLCRP site can be opened, which prompts the GP to do recommended investigations prior to making the referral. The GP Liaison Team (a team of two GPs and administrative staff) at the Townsville University Hospital supervised the implementation of TLCRP as they would with other HealthPathways.

Given the barriers to health care in the rural and remote context of Australia, understanding the experiences of regional, rural and remote users of lung cancer referral pathways is important for quality improvement efforts [17,18,19,20,21]. With any newly implemented health program, it is important that periodic evaluations are conducted to assess intervention effectiveness and inform iterative improvement efforts [22,23,24,25]. Qualitative studies evaluating implementation of various care pathways have been published from Australia, New Zealand, and the United Kingdom [9,10,16,22,23,25,26]. However, none of these studies specifically considered a referral pathway for people with suspected lung cancer in a regional or rural setting.

This study is part of a broader project exploring the implementation of the TLCRP and its impact on patient outcomes, including timeliness of care [Table 1]. Evaluation of patient and carer experience of TLCRP has been published [27]. This paper reports on clinicians’ experiences and perspectives about the TLCRP, providing an important user perspective about its sustainability.

**Table 1:**
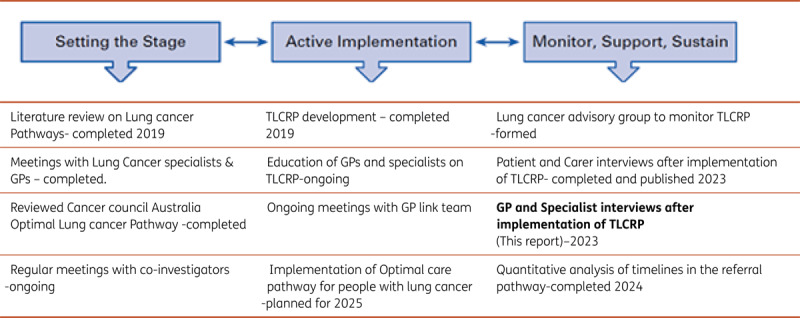
Overall project diagram. Strategic implementation framework for the project. Adapted from Mitchell S et al, 2017, Journal of Oncology Practice [29].

## Methods

### Study design and setting

This was a descriptive qualitative study nested within a larger project evaluating TLCRP, utilising a broader implementation science framework [28,29]. Participants included GPs in the health service region and specialists working at the local tertiary referral hospital.

The ‘Strategic Implementation Framework’ arranges implementation strategies along the continuum of change and this study is part of the monitoring and sustainability stage [29]. Various stages of this framework are interconnected and cyclical, so that the Active Implementation phase may occur alongside the Monitoring phase [29]. The implementation outcomes evaluated by this study were acceptability, uptake and awareness of TLCRP.

### Recruitment

Recruitment of participants followed two different but parallel pathways, GPs and specialists. GPs were identified via an address book located on the hospital intranet. All the GPs in the Townsville Hospital and Health Service (THHS) region were eligible to participate in the study. A purposive approach to recruitment of the sample was conducted by the PI and another member of research team (G K). Initially, an invitation to participate in the study was mailed to all the GP practices in the THHS. However, possibly due to the COVID-19 outbreak, t only few GPs responded to this invitation. The PI and GK then contacted individual GPs by telephone and invited them to participate. We purposively sampled the GPs to construct a maximum variation sample that aimed to include GPs with varying location/rurality. All the GPs who agreed to participate were included in the study. We were aiming to recruit about 15 GPs and stopped recruitment once we had interviewed 14 GPs. The timeline for recruitment of GPs was from October 2021 to May 2022. Although the final number in the maximum variation sample was decided by the number of GPs who agreed to participate, we aimed to recruit total of 15 GPs. GPs were emailed the Participant Information and Consent Form (PICF). They returned it to PI by email after signing.

All the hospital specialists who manage lung cancer (Respiratory physicians (N = 4), Thoracic surgeons (N = 2), Radiation oncologists(N = 2) and medical oncologists(N = 3)) were invited by PI to participate in the study. The timeline of recruitment of specialists was from February 2022 to May 2022. Of the specialists who agreed to participate, the PI selected the participants so that all specialities were represented. None of the Radiation oncologists agreed to participate. The specialists were then provided with the PICF. Once the consent was obtained PI contacted them to arrange suitable time and place for the interview.

### Data collection

The interviews were conducted by the principal investigator (ZO) and another member of the research team (GK), after obtaining written informed consent from participants. The data collection and analysis were done in parallel for both GP and specialist interviews. There were separate interview guides for GPs and specialists (Supplementary material 2). The questions for the interview guide were constructed by the investigators informed by knowledge of the relevant literature and the theoretical framework used through multiple meetings. After revisions, piloting for content validity occurred by interviewing a GP and a medical registrar. The questionnaires were modified after the pilot interviews. The interview guide contained questions on awareness and use of TLCRP, barriers to use and employing telehealth in the referral process (Supplementary material 2). The initial interview questions were open-ended, to facilitate an inductive approach to clinician experiences while not leading participants. Follow-up probing questions were used to elicit deeper responses. The interviews were conducted face-to-face or by telephone (as indicated by participant choice), at a time convenient to the participant. Average duration of the GP interviews were about 30 minutes, and the average duration of specialist interviews were about 20 minutes. Once we were not obtaining any newer information from the GP interviews, recruitment was stopped. This was the deciding point that informed saturation. Since there were only few lung cancer specialists in the hospital, the PI interviewed all the specialists who agreed to participate in the study. All the interviews were audio-recorded and transcribed verbatim, through an external transcribing service to facilitate later qualitative analysis.

### Analysis

An iterative inductive thematic analysis [30] of transcripts was used to derive key codes and themes regarding participant experiences and perceptions. Coding and thematic analysis were performed within NVivo12 software (QSR international, Melbourne, Australia), initially by the primary investigator (ZO) and repeated by another member of the research team (AB), to improve qualitative rigour. Both investigators undertook a preliminary coding exercise, where a code was applied to every comment made by the participants. Each code was a descriptive phrase indicating the theme of the content. This was revised and refined several times by the coders independently and then collaboratively. The results were then discussed in meetings with the other researchers. The final codes were considered subthemes, which were then grouped together under two major themes (Table 4). COREQ (Consolidated criteria for reporting Qualitative research) checklist was used to report the results [31] (Supplementary material 3).

Ethical approval of this study was obtained from the Hospital Human Research Ethics Committee (HREC/2020/QTHS/58635).

## Results

### Descriptive findings

Twenty interviews were conducted, 14 with GPs and six with specialists across disciplines of respiratory medicine, oncology, and cardiothoracic surgery. All the specialists practiced in the public hospital. Of the GPs, 11 were practicing in the regional locality of Townsville and three practiced in surrounding rural areas (GP characteristics can be found in Table 2 and specialist characteristics in Table 3).The years of general practice experience varied from one to 31 years. The reported average number of people seen per year with suspected lung cancer by each GP ranged from one to 10.

**Table 2:** GP characteristics.


GP	YEARS AS GP	REGIONAL (MMM 2 &3) RURAL & REMOTE (MMM 4–7) *	AWARE OF TLCRP Y = YES N = NO	USED TLCRP Y = YES N = NO	WILL USE TLCRP IN THE FUTURE. Y = YES N = NO

1	7	Regional	Y	Y	Y

2	31	Rural	N	N	N

3	12	Regional	Y	N	Y

4	15	Regional	Y	N	Y

5	5	Rural	Y	Y	Y

6	11	Regional	Y	Y	Y

7	8	Regional	N	Y	Y

8	20	Rural	Y	N	Y

9	14	Regional	Y	N	Y

10	11	Regional	Y	Y	Y

11	3	Regional	Y	Y	Y

12	12	Regional	Y	N	Y

13	5	Regional	Y	N	N

14	1	Regional	Y	Y	Y


*MMM (Modified Monash Model) to classify rurality in Australia.

**Table 3:** Specialist characteristics.


	SPECIALITY	YEARS OF EXPERIENCE IN AUSTRALIA	IMPROVEMENT IN REFERRAL PROCESS AFTER IMPLEMENTATION OF TLCRP

1	Respiratory medicine	17	yes

2	Respiratory medicine	4	yes

3	Respiratory medicine	2	yes

4	Medical oncology	11	yes

5	Medical oncology	10	yes

6	Cardiothoracic surgeon	6	yes


Of the 14 GPs, 12 were aware of the TLCRP and seven had used it. Twelve GPs reported that they will use the TLCRP next time they see a patient with suspected lung cancer.

### Thematic findings

Interview analysis identified two major themes and several sub-themes (Table 4).

**Table 4:** Themes.


MAJOR THEMES	SUB-THEMES

Variation in uptake of TLCRP	Awareness and use of TLCRP Benefits of TLCRP Barriers to implementation of TLCRP

Enhancing coordinated care and communication	Co-ordination of patient care between GPs and specialists Integration of telehealth in TLCRP Support for use of TLCRP


### Awareness and use of TLCRP

Most GPs were aware of *Townsville HealthPathways* and used them as part of Smart Referrals.

*“we’re using the HealthPathways more as part of the Smart Referral. It is incorporating the pathway in that Smart Referral anyway.” (Male GP, regional, 20 yrs. experience)*

But some were unaware that there was a specific referral pathway for lung cancer (the TLCRP). Since some of the GPs see only a few people with suspected lung cancer per year, they did not see a need to use the TLCRP. Use of the TLCRP was more common among more junior GPs and GP trainees. Some GPs did not reference the pathway unless they had a specific question about management of a patient. Hospital specialists had become aware of TLCRP during the focus group meetings for implementing TLCRP.GPs found TLCRP to be user-friendly and easily accessible, but one GP mentioned that the follow-up section was superfluous.

*“But the follow-up section is a bit long. Just talking about alcohol and exercise, nutrition. So, that might be superfluous.” (Female GP, regional, 4 yrs. experience)*

### Benefits of TLCRP

Many GPs mentioned that TLCRP streamlined the referral process of a person with suspected lung cancer and encouraged appropriate use of resources. They used TLCRP as a step-by-step guide to do appropriate investigations and referrals for people with suspected lung cancer. GPs also used the information provided in the TLCRP on primary prevention of lung cancer, assessing asbestos exposure registry and educating patients. Once a patient was diagnosed with lung cancer, GPs used the TLCRP HealthPathways information for advice on supportive care and patient education.

*“I use it [TLCRP] more as a reference. It’s very thorough when looking at early signs and symptoms, looking at risk factors. It just walks you through all the investigations to do, besides the CT scan. It is very handy from that respect, just to print out information about lung cancer.” (Female GP, regional, 4 yrs. experience)*

GPs reported that once they started using the referral pathway and did all the necessary investigations, fewer of their referrals were rejected, resulting in earlier specialist appointments for their patients. They felt their patients benefited via more rapid assessment by specialists and fewer duplicated investigations.

*“It [following the TLCRP] will avoid rejections. That’s the main part. Like, we usually get a lot of rejections asking us to do this investigation and re-refer again. So, it avoids that rejection of referrals a lot, because we know what to do before we send [the patient] to the specialist.” (Female GP, regional, 2 yrs. experience)*.

Hospital specialists felt that there had been an improvement in the quality of referrals from GPs, after the implementation of the TLCRP. Oncologists and surgeons reported seeing fewer undiagnosed lung cancer patients. These clinicians felt that, prior to TLCRP implementation, more patients used to be directly referred to oncology and surgical clinics by GPs, leading to duplication of investigations and unnecessary delays in patient care.

*“I don’t have to see unnecessarily undiagnosed patients. Patients also have a certainty of where they’re heading, what they’re doing, if all the providers are confident of the next step of care and everybody is talking the same thing. So, it all sort of helps them build that trust in the system, and that they feel that all the doctors are communicating with each other.” (Oncologist)*.

GPs were more confident in monitoring lung nodules as per the guidelines in TLCRP rather than referring all of them to the respiratory clinic.

*“I don’t send in the nodules if they just need to be followed up, we can do CTs every three months to follow them up, and they only get sent in if they’re progressing.” (Male GP, rural, 3 yrs. experience)*

GPs also reported updating their knowledge and teaching junior doctors or medical students using Townsville HealthPathways, including TLCRP. The TLCRP was felt to be particularly beneficial for new GPs and GP trainees as it provided them with the information required to manage and refer a person with suspected lung cancer.

### Barriers to implementing TLCRP

Reasons cited by GPs in this study for not using TLCRP included: (i) lack of time to use it; (ii) lack of willingness to change referral patterns; (iii) lack of familiarity with HealthPathways and experience of their utility; and (iv) problems with the ‘Smart Referrals’ system. Many GPs stated that they do not have enough time in consultation to use the HealthPathways website.

*“ I don’t have time to go through the HealthPathways, even if I want to use it” (Male GP, rural, 11 yrs. experience)*

GPs in some rural and remote locations reported unstable internet connections were problematic for using online resources such as TLCRP and some general practices did not have electronic referral systems. GPs tended not use the TLCRP and other HealthPathways if there was a glitch in the ‘Smart Referrals’ system.

*“So, I think a lot of GPs have stopped doing Smart Referrals, which kind of takes you out of the HealthPathway, because they’re all linked together on the same program.” (Female GP, regional, 22 yrs. experience*

In addition, existing referral habits of clinicians were found to be a barrier to adoption of HealthPathways. Many senior GPs appeared to have entrenched referral habits and did not feel that using TLCRP would improve patient care, especially if they saw people with suspected lung cancer infrequently.

*“Having a pathway for a specific disease is very hard for me to navigate. If I only use it once a year, it’ll be forgotten. I am happy with my current referral process and don’t think this pathway is going to make it better ” (Male GP, rural, 30 yrs. experience)*

The specialists did not mention any specific barriers to using TLCRP, but they did not routinely use TLCRP.

### Co-ordination of patient care between GPs and specialists

When discussing the TLCRP, clinicians expressed a need for greater integration of GP and specialist care for people suspected of having lung cancer. While the focus of this project was on TLCRP, GPs still found difficulties for accessing care for their patients with lung cancer after specialist referral was completed. There were sometimes significant delays in respiratory clinic appointments and investigative procedures.

‘*I guess the barriers are still the interface between GPs and the hospital. Shortages in staffing. Sometimes patients can’t afford to pay for the scans privately, and certainly we don’t do lung biopsies privately. So, that’s all got to come from the hospital. So, that’s where the hold-up sometimes is. (Female GP, regional, 22 yrs. experience)*

Respiratory physicians attributed delay in specialist appointments to shortage of doctors in the department and lack of adequate investigative facilities and lack of care co-ordinator.

*“I think the manpower is a big problem where there’s a delay in seeing cancer patients, from a respiratory point of view.” (Respiratory physician)”*

*“ I’m just wondering whether MDT coordinators can actually have a designated job plan to say what are their roles. Then, that’ll be actually helpful”. (Respiratory physician)*

GPs mentioned they get frustrated by delayed feedback from specialists about their patients. The lack of a dedicated lung cancer co-ordinator in Townsville University Hospital was mentioned as one of the causes for delays in patient appointments.

### Integrating tele-health in TLCRP

Telehealth was recommended by many GPs and specialists as a strategy to speed up care under the TLCRP. Clinicians used telehealth to consult with people with suspected lung cancer, to discuss results and their management.

*“Since COVID has started, we have done more telehealth. When they have symptoms, we just tell them, “Hey, look, you need to go and get an X-ray or a CT scan straight away,” and then organize investigations based on that. (Male GP, rural,7 yrs. experience)*

Only a few GP practices had facilities for video-linked consultations and most of them used telephone consultations. All the specialists interviewed had access to good quality video-link facilities. There were varying opinions regarding use of telehealth in the lung cancer referral pathway (TLCRP), but most clinicians agreed that telehealth helps to reduce patient travel and improve their care. Most of the clinicians felt that telephone consultations were not appropriate to break bad news or discuss treatment options.

*“Yeah. I find video-linked consultations are better [than phone consultations], because the patient and the treating physician or surgeon can see each other, and the understanding is better — rather than talking over the phone. Minimise unnecessary travels and unnecessary hospital visits, et cetera”. (Thoracic surgeon)*

### Support for the use of TLCRP

Most GPs felt that they received good support and training for using Townsville HealthPathways from the GP Liaison team at the hospital and the local Primary Health Network. It was also reported that the GP liaison team regularly updated the HealthPathways and that this is one of the reasons for increased uptake of TLCRP.

*” I think Primary Health Network has made that part very easy. They gave us IDs and passwords [for Townsville HealthPathways] and then they also have, like, merchandise and things like that. For example, they have given me a mousepad with ID and password, which is there in front of the computer all the time.” (Male GP, rural, 7 yrs. experience)*

## Discussion

This study provides important insights into barriers and enablers of lung cancer referral pathway adoption in a regional Australian health service. Barriers to adoption of TLCRP included practical and technology issues, lack of time and lack of familiarity and experience with HealthPathways. Enablers to adoption of TLCRP were the timely education and training of GPs, the usefulness of TLCRP in patient management and presence of technical support to use TLCRP.

The setting of this study is in north Queensland, servicing regional, rural and remote locations. This setting might raise different issues in terms of access, timeliness and patient preferences compared to the implementation of such a pathway in a metropolitan area [32,33]. Few studies have been done on referral pathways in rural and regional areas. Like other studies, we found that implementation of TLCRP has had a positive impact on how GPs refer lung cancer patients [10,22,25,34]. Most clinicians who participated in this project felt that TLCRP streamlined and improved the quality of GP referrals, resulting in better care for people suspected with lung cancer.

Increased awareness among clinicians was identified as essential to improve uptake of health pathways in other Australian studies [22,35]. Lack of awareness among some participants in this study about TLCRP and its usefulness was found to be a barrier to uptake. This indicates a need to consider more and alternative methods of reaching primary health care clinicians to raise awareness (in addition to those successfully employed to date). Among those GPs that did not routinely use TLCRP, this was most often due to difficulty in accepting change to their workplace routines. Entrenched routines as barriers to adopting the use of health pathways has been identified previously [4,36]. Further exploration of reluctance to use TLCRP and other HealthPathways is required.

We found that lack of integration with existing GP referral systems, inability to create electronic referrals directly from within HealthPathways and the busy, time-pressured nature of general practice were barriers to using TLCRP; results mirrored in a region of New South Wales, Australia [10]. A robust and secure electronic-referral system alongside HealthPathways is key to communication at the interface between GPs and specialists [37]. In Canterbury, New Zealand, the health pathways are integrated with the local e-referral system and this may have facilitated uptake [38]. Many GPs who were interviewed reported not using the TLCRP and other HealthPathways if there were problems with the Smart Referrals portal. Findings of the present study suggest that mechanisms to better incorporate HealthPathways into GPs’ existing routine practice may be helpful. In particular, better integration of HealthPathways with Smart Referrals may improve the uptake of TLCRP and other HealthPathways [39].

A more unique finding of this study is that junior GPs (with experience less than five years) more commonly use the TLCRP compared to senior GPs. There are several potential explanations for this. One is that new GPs are more eager to learn about navigating the health care system and are more comfortable to use novel software [40,41]. Whereas, more experienced GPs, accustomed to a certain referral pattern for lung cancer for many years did not feel the need to change routine practice. This difference in uptake may suggest that different implementation strategies may be required for senior GPs compared to junior GPs.

Few, if any studies have evaluated clinicians’ experience of using telehealth in a lung cancer referral pathway. Telehealth became part of routine clinical practice for most clinicians during the COVID-19 pandemic [42] when this project was conducted. Most of the GPs and specialists were using telehealth to consult patients with suspected lung cancer and arrange necessary investigations required for TLCRP. We found that, quite appropriately, clinicians were not comfortable to discuss a new cancer diagnosis or break bad news using telehealth. But, if face to face consultation was not possible, they would use telehealth, but preferred video consultation over telephone consultation.

## Implications for future research and practice

This study identified several barriers and enablers to using TLCRP by the clinicians working in a regional and rural health service. Many GPs reported not using TLCRP and other HealthPathways due to lack of time. Time pressure is one of the common barriers to adherence to guidelines by GPs [36]. A shortage of GPs in regional and rural areas in Australia could be one of the reasons why GPs feel time pressures. Another problem faced by regional health services is the rapid turnover of GPs [5,43] thus many new GPs may lack awareness of ‘Townsville HealthPathways’ or TLCRP. Health administrators must continue to work to overcome these deficiencies by increasing incentives for GPs to work in regional and rural areas and providing thorough orientation to the systems and supports available for new workforce arrivals. GPs also need to have more funded time in consultations to adequately evaluate patients with suspected lung cancer and appropriately use TLCRP.

Although this study concerned perceptions and experiences of the TLCRP, clinicians still discussed delays in care that were more related to specialist workforce. Even if an appropriate referral has been completed by the GP, there can be delays in appointments to respiratory clinic due to shortages in staffing, inadequate resourcing such as investigative facilities, or lack of co-ordination between various specialists. Increasing the number of respiratory specialists, providing more radiology and pathology facilities and employing specialist lung cancer nurses and lung cancer care coordinators may help to reduce these delays.

Many clinicians in this study proposed increased use of telehealth to reduce the delays in the process as prescribed by the TLCRP. People residing in rural and remote regions can be quickly reviewed by GPs and specialists through telehealth and appropriate investigations arranged [44]. Improving tele-health facilities and internet connection in rural and remote clinics can reduce delays and improve the uptake of TLCRP. However, GPs need adequate resourcing, training and exposure in delivering patient care via the various telehealth options. One of reasons for increased awareness of TLCRP is the dedicated GP-liaison team, who support the GPs and update them regularly about the HealthPathways. A dedicated GP liaison team requires ongoing funding to increase and sustain uptake of pathways.

## Limitations of the study

This study evaluates only one health pathway, in a single health service area, so the results may not be generalizable to other types of care pathways in other locations. The responses from clinicians were sometimes on Townsville HealthPathways in general and not specifically on TLCRP. Many of the clinicians knew that the principal investigator was involved in managing lung cancer and this may have influenced some of their responses. We tried to minimize this bias by adhering to the interview guide and assuring participants that their views and perceptions were important to the improvement of services.

## Conclusion

This study provides important information on experiences of a lung cancer referral pathway by clinicians in a regional and rural area. Emerging themes from this study may be used to reduce the barriers and improve uptake of TLCRP and other local health pathways. Improving the uptake of TLCRP may mean that more patients in our region experience a streamlined journey to more timely specialist lung cancer care and improved health outcomes.

## Additional Files

The additional files for this article can be found as follows:

10.5334/ijic.7627.s1 Supplementary material 1. Appendix 1: Summary of the Townsville lung cancer referral pathway.

10.5334/ijic.7627.s2 Supplementary material 2. Implementation and Evaluation of a Referral Pathway for people with Lung cancer in Townsville health service district.

10.5334/ijic.7627.s3 Supplementary material 3. COREQ (COnsolidated criteria for REporting Qualitative research) Checklist.

10.5334/ijic.7627.s4 Supplementary material 4. Implementation and Evaluation of a Referral Pathway for people with Lung cancer in Townsville health service district.

## References

[1] Sung H, Ferlay J, Siegel RL, Laversanne M, Soerjomataram I, Jemal A, et al. Global Cancer Statistics 2020: GLOBOCAN Estimates of Incidence and Mortality Worldwide for 36 Cancers in 185 Countries. CA: A Cancer Journal for Clinicians. 2021; 71(3): 209–49. DOI: 10.3322/caac.21660 33538338

[2] Valery PC, Coory M, Stirling J, Green AC. Cancer diagnosis, treatment, and survival in Indigenous and non-Indigenous Australians: a matched cohort study. The Lancet. 2006; 367(9525): 1842–8. DOI: 10.1016/S0140-6736(06)68806-5 16753487

[3] Afshar N, English DR, Milne RL. Rural–urban residence and cancer survival in high-income countries: A systematic review. Cancer. 2019; 125(13): 2172–84. DOI: 10.1002/cncr.32073 30933318

[4] Jong KE, Smith DP, Yu XQ, O’Connell DL, Goldstein D, Armstrong BK. Remoteness of residence and survival from cancer in New South Wales. Med J Aust. 2004; 180(12): 618–22. DOI: 10.5694/j.1326-5377.2004.tb06123.x 15200358

[5] Verma R, Pathmanathan S, Otty Z, Binder J, Vangaveti V, Buttner P, et al. Delays in lung cancer referral pathways between Rural and Urban patients in North Queensland: A Mixed Methods Study. Intern Med J. 2018. DOI: 10.1111/imj.13934 29660226

[6] Hall SE, Holman CD, Threlfall T, Sheiner H, Phillips M, Katriss P, et al. Lung cancer: an exploration of patient and general practitioner perspectives on the realities of care in rural Western Australia. Australian Journal of Rural Health. 2008; 16(6): 355–62. DOI: 10.1111/j.1440-1584.2008.01016.x 19032208

[7] Evans SM, Earnest A, Bower W, Senthuren M, McLaughlin P, Stirling R. Timeliness of lung cancer care in Victoria: a retrospective cohort study. Medical Journal of Australia. 2016; 204(2): 75. DOI: 10.5694/mja15.01026 26821108

[8] Lung Clinical Expert Group. National Optimal Lung Cancer Pathway and Implementation Guide; 2017.

[9] McGeoch G, McGeoch P, Shand B. Is HealthPathways effective? An online survey of hospital clinicians, general practitioners and practice nurses. N Z Med J. 2015; 128(1408): 36–46. 25662377

[10] Gray JS, Swan JR, Lynch MA, Tay TM, Mackenzie MJ, Wiggers JH, et al. Hunter and New England HealthPathways: a 4-year journey of integrated care. Australian health review: a publication of the Australian Hospital Association. 2018; 42(1): 66–71. DOI: 10.1071/AH16197 28214475

[11] Lee XJ, Blythe R, Choudhury AAK, Simmons T, Graves N, Kularatna S. Review of methods and study designs of evaluations related to clinical pathways. Aust Health Rev. 2019; 43(4): 448–56. DOI: 10.1071/AH17276 30089529

[12] DeMartino JK, Larsen JK. Equity in cancer care: pathways, protocols, and guidelines. Journal of the National Comprehensive Cancer Network. 2012; 10 Suppl 1: S1–9. DOI: 10.6004/jnccn.2012.0164 23042831

[13] Jensen KH, Maina PJ. Cancer pathways are associated with improved long-term survival. Dan Med J. 2015; 62(2). 25634507

[14] van Hoeve J, de Munck L, Otter R, de Vries J, Siesling S. Quality improvement by implementing an integrated oncological care pathway for breast cancer patients. Breast. 2014; 23(4): 364–70. DOI: 10.1016/j.breast.2014.01.008 24582455

[15] <Implementing-HealthPathways-across-Queensland-a-case-study.pdf>.

[16] Blythe R, Lee X, Simmons T, Cox J, McLean K, Barfield J, et al. Economic Analysis of Specialist Referral Patterns in Mackay, Queensland Following HealthPathways Implementation. J Prim Care Community Health. 2021; 12. DOI: 10.1177/21501327211041489 PMC8422816 34477465

[17] Australian Institute of Health and Welfare. Australia’s health 2016; 2016.

[18] Australian Institute of Health and Welfare. Cancer in Australia In brief 2019; 2019.

[19] Underhill C, Bartel R, Goldstein D, Snodgrass H, Begbie S, Yates P, et al. Mapping oncology services in regional and rural Australia. Australian Journal of Rural Health. 2009; 17(6): 321–9. DOI: 10.1111/j.1440-1584.2009.01106.x 19930199

[20] Chen TY, Morrell S, Thomson W, Baker DF, Walton R, Aranda S, et al. Survival from breast, colon, lung, ovarian and rectal cancer by geographical remoteness in New South Wales, Australia, 2000–2008. Aust J Rural Health. 2015; 23(1): 49–56. DOI: 10.1111/ajr.12172 25689383

[21] George M, Ngo P, Prawira A. Rural Oncology: Overcoming the Tyranny of Distance for Improved Cancer Care. Journal of Oncology Practice. 2014; 10(3): e146–e9. DOI: 10.1200/JOP.2013.001228 24667293

[22] Mansfield SJ, Quirk F, von Treuer K, Gill G. On the right path? Exploring the experiences and opinions of clinicians involved in developing and implementing HealthPathways Barwon. Australian health review: a publication of the Australian Hospital Association. 2016; 40(2): 129–35. DOI: 10.1071/AH15009 26691571

[23] Stokes T, Tumilty E, Doolan-Noble F, Gauld R. HealthPathways implementation in a New Zealand health region: a qualitative study using the Consolidated Framework for Implementation Research. BMJ Open. 2018; 8(12): e025094. DOI: 10.1136/bmjopen-2018-025094 PMC6318537 30598490

[24] Bullivant J, Corbett-Nolan A. Clinical audit: a simple guide for NHS Boards & partners. Healthcare Quality Improvement Partnership (HQIP) London; 2010.

[25] Goddard-Nash A, Makate M, Varhol R, Quirk F, Larsen R, McGeoch G, et al. Evaluation of HealthPathways: an appraisal of usage, experiences and opinions of healthcare professionals in Australia and New Zealand. Aust Health Rev. 2020; 44(4): 590–600. DOI: 10.1071/AH19214 32693906

[26] Senanayake S, Abell B, Novick M, Exley H, Dolejs W, Hutchinson K, et al. Impact and outcome evaluation of HealthPathways: a scoping review of published methodologies. J Prim Health Care. 2021; 13(3): 260–73. DOI: 10.1071/HC21067 34588110

[27] Otty Z, Brown A, Larkins S, Evans R, Sabesan S. Patient and carer experiences of lung cancer referral pathway in a regional health service, a qualitative study. Intern Med J. 2023. DOI: 10.1111/imj.16022 36710377

[28] Aarons GA, Hurlburt M, Horwitz SM. Advancing a conceptual model of evidence-based practice implementation in public service sectors. Adm Policy Ment Health. 2011; 38(1): 4–23. DOI: 10.1007/s10488-010-0327-7 21197565 PMC3025110

[29] Mitchell SA, Chambers DA. Leveraging Implementation Science to Improve Cancer Care Delivery and Patient Outcomes. Journal of Oncology Practice. 2017; 13(8): 523–9. DOI: 10.1200/JOP.2017.024729 28692331 PMC5555033

[30] Nowell LS, Norris JM, White DE, Moules NJ. Thematic Analysis: Striving to Meet the Trustworthiness Criteria. International Journal of Qualitative Methods. 2017; 16(1): 1609406917733847. DOI: 10.1177/1609406917733847

[31] Tong A, Sainsbury P, Craig J. Consolidated criteria for reporting qualitative research (COREQ): a 32-item checklist for interviews and focus groups. Int J Qual Health Care. 2007; 19(6): 349–57. DOI: 10.1093/intqhc/mzm042 17872937

[32] Hall SE, Holman CD, Threlfall T, Sheiner H, Phillips M, Katriss P, et al. Lung cancer: an exploration of patient and general practitioner perspectives on the realities of care in rural Western Australia. Aust J Rural Health. 2008; 16(6): 355–62. DOI: 10.1111/j.1440-1584.2008.01016.x 19032208

[33] Emery JD, Walter FM, Gray V, Sinclair C, Howting D, Bulsara M, et al. Diagnosing cancer in the bush: a mixed methods study of GP and specialist diagnostic intervals in rural Western Australia. Family Practice. 2013; 30(5): 541–50. DOI: 10.1093/fampra/cmt016 23699107

[34] Akehurst J, Sattar Z, Gordon I, Ling J. Implementing online evidence-based care pathways: A mixed-methods study across primary and secondary care. BMJ Open. 2018; 8(12): e022991. DOI: 10.1136/bmjopen-2018-022991 PMC6318508 30598485

[35] Gill SD, Mansfield S, McLeod M, von Treuer K, Dunn M, Quirk F. HealthPathways improving access to care. Aust Health Rev. 2019; 43(2): 207–16. DOI: 10.1071/AH17090 29415799

[36] Lugtenberg M, Burgers JS, Besters CF, Han D, Westert GP. Perceived barriers to guideline adherence: A survey among general practitioners. BMC Family Practice. 2011; 12(1): 98. DOI: 10.1186/1471-2296-12-98 21939542 PMC3197492

[37] McGonigle L, McGeoch G. The Canterbury pathway to integrated care, warts and all. International Journal of Integrated Care. 2017; 17: 449. DOI: 10.5334/ijic.3769

[38] Timmins N, Ham C. The quest for integrated health and social care. The Kings Fund; 2013 2013/09/12/.

[39] Nabelsi V, Lévesque-Chouinard A, Liddy C, Dumas Pilon M. Improving the Referral Process, Timeliness, Effectiveness, and Equity of Access to Specialist Medical Services Through Electronic Consultation: Pilot Study. JMIR Med Inform. 2019; 7(3): e13354. DOI: 10.2196/13354 31293239 PMC6652123

[40] Devitt N, Murphy J. A survey of the information management and technology training needs of doctors in an acute NHS trust in the United Kingdom. Health Information & Libraries Journal. 2004; 21(3): 164–72. DOI: 10.1111/j.1471-1842.2004.00492.x 15318914

[41] Xu J, Hicks-Roof K, Bailey CE, Hamadi HY. Older and Wiser? The Need to Reexamine the Impact of Health Professionals Age and Experience on Competency-Based Practices. SAGE Open Nurs. 2021; 7: 23779608211029067. DOI: 10.1177/23779608211029067 34368438 PMC8312189

[42] Chazan G, Franchini F, Alexander M, Banerjee S, Mileshkin L, Blinman P, et al. Impact of COVID-19 on cancer service delivery: results from an international survey of oncology clinicians. ESMO Open. 2020; 5(6): e001090. DOI: 10.1136/esmoopen-2020-001090 33262203 PMC7709494

[43] Russell DJ, Wakerman J, Humphreys JS. What is a reasonable length of employment for health workers in Australian rural and remote primary healthcare services? Australian health review: a publication of the Australian Hospital Association. 2013; 37(2): 256–61. DOI: 10.1071/AH12184 23497824

[44] Sabesan S, Larkins S, Evans R, Varma S, Andrews A, Beuttner P, et al. Telemedicine for rural cancer care in North Queensland: bringing cancer care home. The Australian Journal Of Rural Health. 2012; 20(5): 259–64. DOI: 10.1111/j.1440-1584.2012.01299.x 22998200

