# Supplementary material for: Clinicians’ Experiences and Perspectives about a New Lung Cancer Referral Pathway in a Regional Health Service
Source: Int J Integr Care. 2024 Apr 4;24(2):3. doi: 10.5334/ijic.7627 (PMC11012087; doi:10.5334/ijic.7627)
Supplement: Supplementary material 1. — Appendix 1: Summary of the Townsville lung cancer referral pathway. [file ijic-24-2-7627-s1.pdf]

Appendix 1: Summary of the Townsville lung cancer referral pathway

Lung Cancer: Background

About lung cancer

Assessment

Practice point

Have high index of suspicion

1. If symptomatic, screen for lung cancer.
2. Consider lung cancer if:
    - unexplained symptoms and signs for > 3 weeks duration, or
    - risk factors.
3. Investigations:
    -

Management

1. If any red flags, request acute respiratory assessment (with CT chest with contrast arranged if it doesn't delay referral).
2. If a likely primary pulmonary nodule is visible on chest X-ray or CT scan, request non-acute respiratory assessment (mark as urgent) for tissue diagnosis and staging.
3. Request non-acute respiratory assessment if imaging shows:
4. If a tissue biopsy confirms lung cancer and CT chest shows a lung mass, request non-acute cardiothoracic surgery assessment if not already organized.
5. If consolidation seen on chest X-ray, treat as pneumonia and repeat imaging in 4 weeks.
6. If any solid nodule < 6 mm present in patient with risk factors , repeat CT chest with contrast in 12 months.

Follow-up

A treatment summary and follow-up care plan will be provided after initial treatment (request if not provided).

1. Ensure the patient is making healthy lifestyle choices regarding:
2. Provide ongoing support of patient and family and psychosocial management throughout the course of cancer treatment and after.
3. Consider Cancer Support Services, addressing:
4. Offer patient information about:
    - Lung Foundation resources.

- referral to a lung cancer support nurse for patient and family support.

5. Consider Advanced Care Planning (ACP).
6. If required, arrange palliative care.

Information

For health professionals

For patients
