# Supplementary material for: Clinicians’ Experiences and Perspectives about a New Lung Cancer Referral Pathway in a Regional Health Service
Source: Int J Integr Care. 2024 Apr 4;24(2):3. doi: 10.5334/ijic.7627 (PMC11012087; doi:10.5334/ijic.7627)
Supplement: Supplementary material 2. — Implementation and Evaluation of a Referral Pathway for people with Lung cancer in Townsville health service district. [file ijic-24-2-7627-s2.pdf]

Implementation and Evaluation of a Referral Pathway for people with Lung cancer in Townsville health service district.

Semi-structured Interview guide for GPs

Introduction: The purpose of this interview is to gather your thoughts on the Townsville Lung cancer Referral Pathway. This interview will take about 20 minutes. The interview will be audiotaped and transcribed to assist with later data analysis. All names will be removed from typing and your opinions will remain anonymous. Are you ready to start the interview now?

Demographics: s c

- Do you practice in a rural area? palm isl Which town (Postcode)?
- How many patients with suspected lung cancer do you see in a year, on average? ________
- How long have you been practicing in Australia _____(years)__ and in THHS_______(years)?

1. Are you aware of the Lung Cancer Referral Pathway available in the 'Townsville HealthPathways' website? Yes / No

   And have you used it? Yes / No

   ☐ **If no**-
   What is your current referral pattern for patients with suspected Lung cancer?
   Prompts-
   - ☐ *what investigations do you do prior to referring them to a specialist? ?CXR/CT scan*
   - ☐ *Which specialist do you refer the patients to and why?*
   - ☐ *How long does it take to get the patient to specialist appointment-in public & in private*?
   - ☐ What is the reason for not using the referral pathway?

(Go to Q 6)

   ☐ **If yes**- How did you become aware of the pathway? (note all that are relevant)
   - o THHS newsletter
   - o Primary Health Network (PHN)
   - o Colleagues
   - o During GP training

2. What has been your experience of using the Lung Cancer Referral Pathway?
   - o Has anything changed in your practice to allow you to follow the Lung Cancer Referral Pathway?
     - ☐ No
     - ☐ Yes, please describe changes required: ____________________

Prompts –

How quickly can you refer a patient suspected lung cancer to respiratory clinic?

Do you send an urgent referral or call the specialist/ registrar?

3. In your opinion, are there benefits to using the Townsville Lung Cancer Referral Pathway?
   If yes:

3a. What are the benefits of using the referral pathway for yourself as a clinician?

____

____

Prompts:

- ☐ *Better assistance with patient navigation, getting all necessary investigations prior to specialist appointment*
- ☐ *earlier appoints for their patients with specialist,*
- ☐ *better feedback from specialists-how prompt is it?*

3b. Are there benefits for patients by using the referral pathway?

Prompts only- -timely appointments, earlier diagnosis, more information about their condition, less anxiety about their lung cancer journey.

____

____

4. What are the barriers to following the Townsville Lung Cancer Referral Pathway?

(Clarification, if needed: what makes it difficult to follow the TLCRP?)

(Prompts)

- ☐ *Needs to login using a password*
- ☐ *difficulty in getting imaging prior to referral,*
- ☐ *difficulty in appointment with specialists,*
- ☐ *patients finding it difficult to travel*
- ☐ *Any difference between public and private patients?*

5. Do you have any suggestions to improve the Townsville Lung Cancer Referral Pathway?

prompts

- *Suggestions to provide better and tailored information.*
- *Suggestions to improve co-ordination between GPs and specialists- e.g., use of lung cancer care co-ordinators / nurse navigators.*
- *Prompt communication from specialists*
- *Electronic referral*

6. We are also interested in including tele-health consultations in the lung cancer referral pathway. What is your experience of using tele-health to communicate with people being investigated for lung cancer?

____

____

____

6a. What is your opinion about using video-linked consultations or telephone consultations to communicate with people with possible lung cancer or their carers?

____

____

____

____

Prompts:
What is your opinion about use of video-linked consultations to discuss sensitive issues or break bad news?
What is your opinion about use of telephone consultations to discuss sensitive issues or break bad news?

7. Do you have any other comments you would like to add about the Townsville Lung Cancer Pathway before we conclude this interview?
   ____
   ____
   ____
   ____
